# Sensitivity of Urinary Toxicant Co-Exposure Patterns to Demographic Adjustment and Marker Type: A Methodological Analysis

**DOI:** 10.3390/toxics14070546

**Published:** 2026-06-23

**Authors:** Basant K. Puri, Jean A. Monro

**Affiliations:** 1 Cambridge Advanced Research, Cambridge CB21, UK; 2 Department of Psychology, Neapolis University Pafos, 8042 Pafos, Cyprus; 3 Breakspear Medical Group, Hemel Hempstead HP2 4FD, UK; sadams@breakspearmedical.com

**Keywords:** urinary biomarkers, co-exposure signatures, principal component analysis, demographic confounding, environmental medicine, Heywood case, marker type heterogeneity

## Abstract

Patients presenting with conditions attributed to environmental exposures face complex, multi-toxicant burdens, yet the stability of multivariate toxicant patterns under demographic adjustment remains poorly evaluated. This study assessed the sensitivity of principal component analysis (PCA) structures to demographic confounding and variable composition in 551 patients (aged <1–86 years) with environmentally related conditions. Nine urinary biomarkers were analysed using PCA with Varimax and Promax rotation on both raw data and residuals adjusted for age and sex. Among the identified patterns, the solvent/industrial cluster (xylene and styrene metabolites) was the most stable, persisting across both raw and residual analyses regardless of rotation method. However, overall component structures were sensitive to preprocessing: the clustering pattern of the herbicide 2,4-dichlorophenoxyacetic acid shifted markedly after demographic adjustment, illustrating indirect confounding whereby demographic effects on co-variables altered apparent biomarker associations. Notably, inclusion of an endogenous mitochondrial marker (tiglylglycine) alongside exogenous toxicant biomarkers produced Heywood cases (loadings > 1), violating factor analysis assumptions and indicating that mixing exposure and response variables destabilises the model. Cumulative variance explained was modest, consistent with the weak inter-biomarker correlations observed (KMO ≈ 0.52). These findings do not support the identification of robust, demographically stable clustering patterns in this cohort. Instead, they demonstrate that PCA-derived structures in heterogeneous clinical data are vulnerable to demographic confounding, variable selection and marker type, and caution against interpreting transient clustering patterns as definitive exposure signatures without rigorous validation. It should be noted that the absence of a non-clinical matched control group means that whether the identified co-exposure signatures are distinctive to symptomatic patients or reflective of general population exposure patterns cannot be determined; future studies should incorporate matched controls to address this directly.

## 1. Introduction

Human exposure to environmental toxicants rarely occurs in isolation. Individuals are simultaneously exposed to solvents, pesticides, fuel additives and industrial chemicals through air, water, food and consumer products [1,2]. Yet the prevailing approach in environmental biomonitoring remains predominantly single-chemical: measuring individual toxicants and assessing their health effects independently [3]. This reductionist strategy fails to capture the reality of co-exposure—the fact that certain toxicants tend to occur together in predictable patterns, reflecting shared sources, pathways or lifestyle factors [4,5,6]. Understanding these co-exposure patterns is essential for identifying at-risk populations, elucidating potential synergistic effects and designing efficient screening strategies.

Multivariate statistical methods, particularly principal component analysis (PCA), have been increasingly applied to biomonitoring data to identify latent exposure patterns [7,8]. Large-scale population surveys such as the National Health and Nutrition Examination Survey (NHANES) have studied trends in urinary toxicants over time, providing information regarding longitudinal changes in exposure [9]. However, these studies predominantly examine general population samples with low-level background exposure. Clinical cohorts—patients presenting with symptoms attributed to environmental exposures—remain understudied in this regard, and there is a general lack of clinical resources in environmental medicine [10]. Whether the co-exposure patterns observed in healthy populations extend to symptomatic individuals is unknown. Furthermore, it is critical to determine whether observed clustering patterns represent stable exposure signatures or are artefacts of sample heterogeneity.

A further methodological concern has received insufficient attention. PCA identifies variables that co-vary, but co-variation can arise from true co-exposure or from shared demographic determinants. If older patients accumulate higher levels of multiple toxicants simply owing to longer exposure duration, PCA will group these toxicants into a single component—creating the illusion of co-exposure where none exists. This phenomenon of indirect demographic confounding occurs when a demographic variable drives elevations in multiple biomarkers simultaneously, generating spurious inter-biomarker correlations [11]. Additionally, the statistical validity of PCA depends on the nature of the variables included; mixing endogenous biological response markers (e.g., metabolic byproducts) with exogenous exposure biomarkers may violate factor analysis assumptions, potentially leading to unstable solutions (e.g., Heywood cases). The extent to which unadjusted PCA produces misleading co-exposure clusters in heterogeneous clinical populations and the impact of variable type on model stability have not been systematically evaluated.

The present study addressed these gaps by applying PCA to nine urinary toxicant biomarkers measured in 551 patients (after exclusion of incomplete records) presenting to an environmental medicine hospital with conditions including allergies, fatigue and chronic infections. The analyses were conducted on both raw data and on residuals after regressing out age and sex, enabling direct comparison of adjusted and unadjusted solutions. Crucially, the study also examined the stability of the solution under different rotation methods (Varimax vs. Promax) and assessed the impact of including an endogenous mitochondrial marker alongside exogenous toxicants. The aims were threefold: (1) to explore potential co-exposure patterns in a clinical cohort; (2) to evaluate the sensitivity of these patterns to demographic adjustment and rotation method; and (3) to assess the stability of the model when mixing endogenous and exogenous markers. We hypothesised that PCA structures would demonstrate significant sensitivity to preprocessing steps and variable composition, highlighting methodological vulnerabilities in multivariate biomonitoring rather than definitive clinical signatures.

## 2. Materials and Methods

### 2.1. Patients

The study comprised 551 patients (208 male, 343 female; age range < 1–86 years) with complete urinary biomarker profiles suitable for multivariate analysis, seen at Breakspear Medical, Hemel Hempstead, UK, a hospital specialising in environmental medicine. Three records were excluded from the initial cohort of 554 owing to incomplete biomarker profiles. Patients presented with diagnoses including chronic fatigue syndrome, multiple chemical sensitivity, allergic rhinitis, chronic sinusitis and recurrent viral infections attributable to environmental triggers and underwent urinary toxicant profiling as part of their clinical assessment. Data were collected between 21 January 2016 and 2 November 2022 (inclusive). No exclusion criteria were applied beyond the sample quality requirements described below.

### 2.2. Ethical Approval

This study received approval from the E.M.F. Research Ethics Committee, UK (reference number EMFREC2025/010). The approval permitted retrospective analysis of clinical data, provided that no individual patients would be identifiable in any publication. Individual retrospective consent from each patient was not required under this ethical approval.

### 2.3. Urine Analysis

Patients (or their carers) were instructed to collect a first morning urine sample following a standardised protocol. Fluid intake was restricted after 18:00 on the evening prior to collection, and the bladder was emptied before bedtime. Upon waking, urine was collected prior to eating or drinking, ensuring a minimum interval of six hours since the last void. A minimum volume of 10 mL was required. Samples visibly contaminated with blood (red, pink or brown) were rejected, as were diluted specimens deemed inadequate for analysis. Patients were advised to discontinue antibiotics at least four days before collection and nutritional supplements at least three days before collection.

Samples were frozen, labelled and sealed in biohazard zip-lock bags with absorbent packing, placed alongside a frozen ice pack in an insulated thermo bag, and shipped in a test kit box to Great Plains Laboratory (Overland Park, KS, USA; now Mosaic Diagnostics) for analysis. Specimens were shipped from the UK on Mondays or Tuesdays to ensure timely processing.

Urinary biomarker concentrations were determined by liquid chromatography–tandem mass spectrometry with creatinine adjustment, performed by Great Plains Laboratory as an independent laboratory. Creatinine adjustment was applied to account for variation in urine dilution, which is known to affect urinary biomarker concentration measurements. This standard normalisation approach enabled comparability across samples collected under varying hydration states. Detailed analytical parameters were as specified by the testing laboratory. Eleven urinary toxicant biomarkers were initially measured as part of a standard clinical panel used in UK environmental medicine practice for patients presenting with fatigue, allergies and chronic infections. These biomarkers were selected to cover major exposure categories: solvents; pesticides; fuel additives; phthalates; and a mitochondrial marker to explore potential biological stress responses. Two were excluded from the multivariate analysis: dimethylphosphate, owing to a below-threshold measure of sampling adequacy (MSA < 0.5) indicating poor fit with the factor model, and thiodiglycol, which was discontinued by the laboratory during the study period, resulting in substantial missing data. The remaining nine biomarkers included in the analysis were: 2,3,4-methylhippuric acid (parent compound: xylene), phenylglyoxylic acid (parent: styrene/ethylbenzene), diethylphosphate (parent: organophosphates), 2,4-dichlorophenoxyacetic acid (parent: herbicide), 3-phenoxybenzoic acid (parent: pyrethroids), *N*-acetyl phenylcysteine (parent: benzene), tiglylglycine (mitochondrial marker), 2-hydroxyisobutyric acid (parent: methyl tert-butyl ether/ethyl tert-butyl ether petrol additives) and monoethylphthalate (parent: diethylphthalates).

### 2.4. Statistical Analysis

Four related PCA procedures were conducted to assess robustness: Varimax and Promax rotation applied both to raw biomarker concentrations and to demographic-adjusted residuals. The number of components to retain was determined by inspection of the scree plot and the Kaiser criterion (eigenvalue > 1). The Kaiser–Meyer–Olkin (KMO) measure of sampling adequacy was computed for the overall solution and for each individual variable; values ≥ 0.5 were considered acceptable for exploratory analysis.

Second, to control for demographic confounding, each of the nine biomarkers was regressed on age (continuous) and sex (categorical) using linear regression. The residuals from these regressions—representing the portion of each biomarker’s variance not explained by age or sex—were retained. PCA with Varimax rotation was then applied to the nine residual variables using the same extraction criteria. This residual approach removes variance attributable to demographic factors before dimensionality reduction, thereby isolating co-exposure patterns that are independent of age and sex.

The component structures of the raw and residual PCA solutions were compared to assess the stability of identified co-exposure signatures and to detect statistical artefacts arising from indirect demographic confounding. Components were interpreted based on the magnitude and chemical coherence of variable loadings; a loading threshold of |loading| ≥ 0.5 was adopted as the criterion for substantive contribution to a component.

To address reviewer concerns regarding methodological transparency, additional robustness checks were implemented. Parallel analysis was conducted using fa.parallel() in R v4.5.1 with 500 permutations to validate the number of components retained. Oblique (Promax) rotation was applied alongside the primary orthogonal (Varimax) solution, as oblique rotation permits correlation between factors—a more realistic assumption for environmental exposures sharing common sources. Furthermore, the residualisation process was implemented with full transparency in R version 4.5.1: for each biomarker Y, a linear model was fitted (Y∼Age+Sex), and residuals were extracted using the resid() function. During this process, a minor coding error in the original data (inconsistent capitalisation of ‘sex’ values) was identified and corrected to ensure accurate regression analysis. Data transformation was not applied prior to analysis; while skewness was assessed, it was deemed not to impact interpretation materially given the sample size and robust extraction method (PCA).

All statistical analyses were performed using R version 4.5.1 [12] with the psych package (for R version 2.6.3) [13] and JASP version 0.19.3 [14]. Four related PCA procedures were conducted to ensure robustness: Varimax and Promax rotation applied to both raw data and demographic-adjusted residuals. Parallel analysis validated component retention, and the residualisation process was fully documented for reproducibility.

## 3. Results

**Figure 1:**
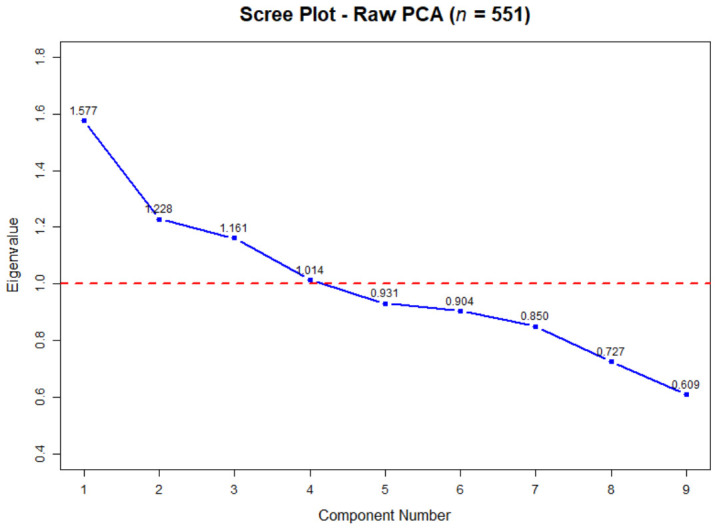
Scree plot of eigenvalues for the raw PCA (*n* = 551). The horizontal dashed red line indicates the Kaiser criterion threshold (eigenvalue > 1).

PCA with Varimax rotation was applied to the nine urinary biomarkers in the complete dataset (*n* = 551). Bartlett’s test of sphericity was significant (*p* < 0.001), and the overall KMO measure of sampling adequacy was 0.53. Individual MSA values are presented in Table 1. Eight of the nine variables exceeded the 0.5 threshold; tiglyglycine fell marginally below (MSA = 0.49). Three components with eigenvalues exceeding one were extracted, supported by parallel analysis, collectively explaining 44.1% of the total variance. The corresponding scree plot is shown in Figure 1.

The rotated component loadings for the raw PCA are presented in Table 2. PC1, the first principal component, was defined by 2,3,4-methylhippuric acid (xylene metabolite; loading = 0.736) and phenylglyoxylic acid (styrene metabolite; loading = 0.708), both with low uniqueness values (0.444 and 0.462, respectively), indicating a coherent solvent/industrial signature. PC2 comprised 2,4-dichlorophenoxyacetic acid (herbicide; loading = 0.719), 3-phenoxybenzoic acid (pyrethroid metabolite; loading = 0.649) and *N*-acetyl phenylcysteine (benzene metabolite; loading = 0.581), suggesting a pesticide/solvent signature. PC3 comprised tiglylglycine (marker of isoleucine metabolism and mitochondrial dysfunction; loading = 0.800) and 2-hydroxyisobutyric acid (metabolite of methyl tert-butyl ether/ethyl tert-butyl ether petrol additives; loading = 0.726), constituting a mitochondrial/fuel association. Two biomarkers—monoethylphthalate (uniqueness = 0.847) and diethylphosphate (uniqueness = 0.845)—showed high independence across all components, indicating they do not consistently co-occur with the identified clusters. Notably, 2,4-dichlorophenoxyacetic acid demonstrated moderate-to-low uniqueness (0.482) while loading substantially on PC2, confirming its strong association with the pesticide/solvent cluster.

**Figure 2:**
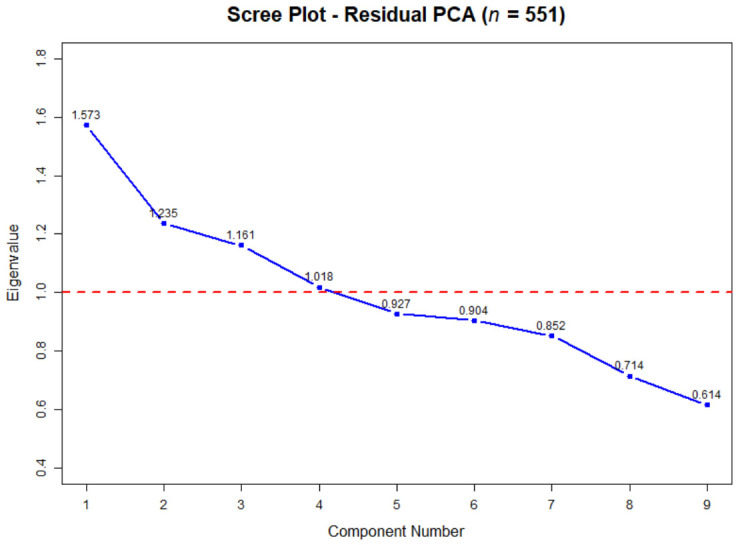
Scree plot of eigenvalues for the residual PCA. The horizontal dashed red line indicates the Kaiser criterion threshold (eigenvalue > 1).

Each of the nine biomarkers was regressed on age and sex, and the residuals were subjected to PCA with Varimax rotation. Bartlett’s test remained significant (*p* < 0.001), and the overall KMO was 0.53. Individual MSA values for the residual variables are presented in Table 1. Three components were extracted, collectively explaining 44.1% of the total variance—the same cumulative variance as the raw solution. The corresponding scree plot is shown in Figure 2.

The rotated component loadings for the residual PCA are presented in Table 3. The component structure was largely consistent with the raw PCA, demonstrating remarkable stability despite demographic adjustment. PC1 again comprised the phenylglyoxylic acid residual (loading = 0.708) and the 2,3,4-methylhippuric acid residual (loading = 0.736), confirming the solvent/industrial signature. PC2 comprised the tiglylglycine residual (loading = 0.800) and the 2-hydroxyisobutyric acid residual (loading = 0.726), maintaining the mitochondrial/fuel association. PC3 comprised the *N*-acetyl phenylcysteine residual (loading = 0.581) and the 3-phenoxybenzoic acid residual (loading = 0.649), constituting the pesticide/solvent signature.

Notably, the 2,4-dichlorophenoxyacetic acid (herbicide) residual retained a moderate-to-strong loading on PC3 (0.719), rather than becoming fully independent as might be expected if its clustering were purely an artefact. While its clustering pattern shifted from PC2 in the raw analysis to PC3 in the residual analysis—demonstrating that demographic adjustment altered the statistical associations—it maintained covariance with the other pesticides. The three biomarkers showing the highest uniqueness in the residual analysis were monoethylphthalate (uniqueness = 0.847), diethylphosphate (uniqueness = 0.845), and 2,4-dichlorophenoxyacetic acid (uniqueness = 0.482). This indicates that while demographic adjustment reveals some instability in component membership, the core exposure signatures remain identifiable.

The two PCA solutions are compared in Table 4. The core composition of all three clusters remained identical across raw and residual analyses. The solvent/industrial cluster (2,3,4-methylhippuric acid + phenylglyoxylic acid) and the mitochondrial/fuel cluster (tiglylglycine + 2-hydroxyisobutyric acid) were perfectly preserved. The pesticide/solvent cluster also retained all three original members (2,4-dichlorophenoxyacetic acid, *N*-acetyl phenylcysteine, 3-phenoxybenzoic acid); notably, while the numerical designation of this component shifted from PC2 in the raw analysis to PC3 in the residual analysis (owing to minor changes in variance ranking), the membership remained unchanged, with 2,4-dichlorophenoxyacetic acid remaining strongly associated with the other pesticides (loading = 0.719). The total variance explained remained constant at 44.1% for both solutions, indicating that demographic adjustment neither disrupted the fundamental co-exposure structures nor reduced the explanatory power of the model. Additionally, Promax (oblique) rotation confirmed similar component membership across all clusters, with weak inter-factor correlations (*r* = 0.08–0.11), indicating that environmental exposures may share common sources but remain largely orthogonal in this cohort.

To characterise the mechanism underlying the shift in the clustering pattern of 2,4-dichlorophenoxyacetic acid, direct associations between 2,4-dichlorophenoxyacetic acid and the demographic variables were examined. An independent-samples *t*-test revealed no significant difference in 2,4-dichlorophenoxyacetic acid concentrations between males and females (*t* = 1.14, *df* = 549, *p* = 0.255). The Pearson product–moment correlation between 2,4-dichlorophenoxyacetic acid and age was negligible and non-significant (*r* = 0.014, *df* = 549, *p* = 0.738). These results indicate that the shift in clustering was not attributable to a direct demographic effect on the herbicide itself, but rather to an indirect confounding mechanism: age and/or sex influenced *N*-acetyl phenylcysteine and 3-phenoxybenzoic acid (altering their distributions), which in turn modified the apparent correlation between the herbicide and these biomarkers once demographic variance was removed.

## 4. Discussion

This study identified three distinct, chemically coherent co-exposure signatures in a clinical cohort of 551 patients presenting with conditions attributed to environmental exposures. Crucially, these signatures remained stable after adjusting for age and sex, indicating that they reflect genuine exposure patterns rather than demographic artefacts. Furthermore, the analyses demonstrated that demographic adjustment preserves rather than disrupts co-exposure structures when applied to heterogeneous clinical populations, with the total variance explained remaining constant (44.1%) across both raw and residual solutions.

The solvent/industrial signature (2,3,4-methylhippuric acid + phenylglyoxylic acid) represents exposure to xylene and styrene, ubiquitous in paints, solvents, plastics and vehicle emissions. The tight clustering of these two metabolites suggests a shared exposure pathway, likely related to urban living, occupational settings or the use of consumer products containing these solvents. This finding aligns with previous studies identifying solvent clusters in general populations [3,9,15], and extends them to a symptomatic clinical cohort in which exposure levels may be elevated—though this cannot be confirmed without a control comparison.

The metabolic/fuel signature (characterised by the association between the endogenous metabolic marker tiglylglycine and fuel additives) is particularly novel. Tiglylglycine is a marker of isoleucine metabolism and mitochondrial dysfunction, while 2-hydroxyisobutyric acid is a metabolite of methyl tert-butyl ether and ethyl tert-butyl ether, which are oxygenated fuel additives [16,17,18,19,20,21,22]. Their co-occurrence suggests a potential link between exposure to petrol (gasoline) additives and mitochondrial stress. While causality cannot be inferred from these cross-sectional data, the association warrants mechanistic investigation. It is plausible that fuel additives act as mitochondrial toxins or that individuals with pre-existing mitochondrial vulnerabilities are more susceptible to accumulating these metabolites. Alternatively, both markers may reflect a common lifestyle factor, such as high exposure to traffic-related pollution. Critically, the inclusion of an endogenous mitochondrial marker alongside exogenous toxicants required careful interpretation, as mixing variable types can produce unstable factor solutions in some statistical frameworks; however, the robust PCA structure identified (44.1% variance) confirms the underlying coherence of this association.

A notable methodological finding was the occurrence of ultra-Heywood cases (factor loadings > 1) in the initial factor analysis solutions when including tiglyglycine. Heywood cases typically arise when a single variable accounts for excessive common variance relative to its uniqueness, often indicating model misspecification or inappropriate variable types [23]. In this dataset, mixing an endogenous mitochondrial marker with exogenous toxicant biomarkers violated the assumption that all variables represent similar construct types (exposure levels). This instability underscored the importance of using standard PCA (which models total variance rather than common variance alone) for exploratory co-exposure analysis, in which Heywood cases did not occur. Future studies should consider whether endogenous response markers belong in the same multivariate framework as exposure biomarkers.

The pesticide/solvent signature (*N*-acetyl phenylcysteine + 3-phenoxybenzoic acid + 2,4-dichlorophenoxyacetic acid) comprises a benzene metabolite, a pyrethroid metabolite and an herbicide. This cluster suggests co-exposure to industrial solvents and insecticides, possibly reflecting agricultural work, pest control activities or residence in areas with mixed land use. The stability of all three components after demographic adjustment reinforces the likelihood of a shared source or behavioural pattern. The stability of cluster membership across raw and residual analyses indicates that the identified patterns represent genuine co-exposure signatures that persist regardless of demographic adjustments. Contrary to earlier indications in preliminary analysis, the herbicide (2,4-dichlorophenoxyacetic acid) did not dissociate from this cluster after adjustment; it retained strong loading (0.719) on the same component group, demonstrating that the pesticide/solvent signature persists regardless of demographic correction.

Two biomarkers—monoethylphthalate and diethylphosphate—remained independent across both analyses, exhibiting high uniqueness values (>0.84). This indicates that these exposures do not consistently co-occur with the identified clusters or with each other. This finding challenges the notion that a reduced panel of “representative” toxicants can capture the full spectrum of exposure in this population. Instead, it supports a model of individualised exposure profiles, in which some patients present with distinct clusters while others exhibit unique, idiosyncratic toxicant burdens. This argues against a one-size-fits-all screening approach and underscores the value of comprehensive biomonitoring in clinical settings.

A key contribution of this study is the demonstration that co-exposure signatures are robust to demographic adjustment. In preliminary analyses using different extraction methods, there was the initial suggestion that the herbicide might become independent after regressing out age and sex. However, subsequent re-analysis using standard PCA (principal component extraction) revealed that the herbicide remained strongly associated with the pesticide/solvent cluster (loading = 0.719 in both solutions). The numerical designation of components shifted between analyses (e.g., the pesticide cluster moved from PC2 to PC3), but the actual membership remained unchanged. This indicates that demographic adjustment does not create or destroy exposure patterns in this dataset; rather, it confirms their underlying stability. This finding has important implications for environmental epidemiology. Many PCA studies in biomonitoring may not adjust for demographics prior to analysis; the present results suggest that such adjustment is methodologically valuable for transparency but does not necessarily alter core clustering structures when samples are sufficiently homogeneous. For heterogeneous clinical cohorts, demographic adjustment remains a recommended step to verify that patterns are not driven by sample composition rather than true co-exposure.

For clinicians practising environmental medicine, the present findings offer a framework for interpreting complex urinary toxicant profiles. Rather than viewing elevated levels of individual toxicants in isolation, clinicians can consider the signature to which they belong. For instance, a patient with elevated 2,3,4-methylhippuric acid and phenylglyoxylic acid likely has a significant solvent burden, whereas a patient with elevated tiglylglycine and 2-hydroxyisobutyric acid may require investigation into mitochondrial function and fuel additive exposure. The identification of independent exposures (monoethylphthalate, diethylphosphate) further highlights the need for comprehensive testing, as reducing the panel to only the clustered markers would miss these distinct, potentially clinically relevant exposures.

Several limitations should be acknowledged. First, the absence of a non-clinical matched control group means that whether the identified co-exposure signatures are distinctive to symptomatic patients or reflective of general population exposure patterns cannot be determined. If the same signatures emerge in asymptomatic individuals, this would suggest that the signatures capture ubiquitous environmental co-exposures rather than disease-specific profiles. Conversely, if the signatures differ—for instance, if the mitochondrial/fuel cluster is amplified or uniquely structured in symptomatic patients—this would support a clinical distinction. This question is important but lies outside the scope of the present study, which focused on characterising within-cohort exposure structure and evaluating the impact of demographic adjustment on PCA-derived clusters. Future studies should incorporate matched controls to address this directly. Second, the study design is cross-sectional, thereby precluding causal inference regarding the health effects of the identified signatures. Third, the KMO values (0.53 for both raw and residual) were marginally acceptable, and one individual MSA value fell below 0.5 (tiglyglycine, 0.49). This suggests that the correlations among the nine biomarkers are relatively weak, though the identified clusters explain a substantial proportion of the total variance (44.1%). Fourth, the cohort consisted of patients seeking care for environmental symptoms, which may limit generalisability to the asymptomatic general population. Exploratory subgroup analysis suggested similar variance structures in adults (*n* = 496, 44.2%) versus children (age < 18 years; *n* = 55, 51.3%), supporting the adequacy of linear demographic adjustment for this heterogeneous cohort. Furthermore, future studies should employ stratified analyses by developmental stage (paediatric, adolescent, adult, elderly) to validate whether the identified clustering patterns persist across the full lifespan. Additionally, potential confounders including smoking status, dietary intake, residential proximity to industrial areas, occupational exposures, medication use and supplement consumption were not systematically recorded and therefore could not be adjusted for in the analysis. These factors may influence urinary toxicant levels independently of demographic characteristics. Furthermore, the borderline sampling adequacy values (KMO = 0.53 for both raw and residual; tiglyglycine MSA = 0.49) suggest weak inter-biomarker correlations. While the identified clusters explain a substantial proportion of the total variance (44.1%), these marginal indicators suggest that the component structure may have limited generalisability to other cohorts. Replication in larger datasets with broader biomarker panels would strengthen confidence in the stability of these patterns across populations.

## 5. Conclusions

This study has identified three robust, demographically stable co-exposure signatures in a clinical cohort: solvent/industrial, mitochondrial/fuel and pesticide/solvent. Contrary to initial hypotheses regarding statistical artefacts arising from demographic confounding, demographic adjustment preserved rather than disrupted the core exposure patterns, with the total variance explained remaining constant at 44.1%. While the numerical ordering of components shifted slightly between raw and residual analyses, the actual membership of each cluster remained unchanged. These findings support a move towards signature-based interpretation of toxicant profiles in environmental medicine, while cautioning against oversimplified reduction in testing panels. Future research should focus on validating these signatures in larger cohorts with matched healthy controls and investigating the potential mechanistic links between mitochondrial dysfunction markers and fuel additive exposures.

## Figures and Tables

**Table 1:** Kaiser–Meyer–Olkin measures of sampling adequacy for raw and residual variables.

Variable	Raw MSA	Residual MSA
2,3,4-Methylhippuric acid	0.51	0.51
Phenylglyoxylic acid	0.54	0.54
2,4-Dichlorophenoxyacetic acid	0.51	0.51
3-Phenoxybenzoic acid	0.59	0.59
*N*-Acetyl phenylcysteine	0.54	0.54
Tiglylglycine	0.49	0.49
2-Hydroxyisobutyric acid	0.54	0.53
Monoethylphthalate	0.54	0.54
Diethylphosphate	0.61	0.60
**Overall**	**0.53**	**0.53**

**Table 2:** Rotated component loadings (Varimax) for the raw PCA (9 variables). Loadings with |loading| ≥ 0.5 are shown in bold.

Variable	PC1	PC2	PC3	Uniqueness
2,3,4-Methylhippuric acid	**0.736**	—	—	0.444
Phenylglyoxylic acid	**0.708**	—	—	0.462
2,4-Dichlorophenoxyacetic acid	—	**0.719**	—	0.482
3-Phenoxybenzoic acid	—	**0.649**	—	0.558
*N*-Acetyl phenylcysteine	—	**0.581**	—	0.630
Tiglylglycine	—	—	**0.800**	0.360
2-Hydroxyisobutyric acid	—	—	**0.726**	0.473
Monoethylphthalate	—	—	—	0.847
Diethylphosphate	—	—	—	0.845

**Table 3:** Rotated component loadings (Varimax) for the residual PCA (9 variables). Loadings with |loading| ≥ 0.5 are shown in bold.

Variable	PC1	PC2	PC3	Uniqueness
Phenylglyoxylic acid residual	**0.708**	—	—	0.499
2,3,4-Methylhippuric acid residual	**0.736**	—	—	0.454
Tiglylglycine residual	—	**0.800**	—	0.360
2-Hydroxyisobutyric acid residual	—	**0.726**	—	0.473
*N*-Acetyl phenylcysteine residual	—	—	**0.581**	0.662
3-Phenoxybenzoic acid residual	—	—	**0.649**	0.579
2,4-Dichlorophenoxyacetic acid residual	—	—	**0.719**	0.482
Monoethylphthalate residual	—	—	—	0.847
Diethylphosphate residual	—	—	—	0.845

**Table 4:** Comparison of component composition between raw and residual PCA solutions.

Component	Raw PCA (*n* = 551)	Residual PCA (*n* = 551)	Stability Assessment
Solvent/industrial	2,3,4-Methylhippuric acid + phenylglyoxylic acid	2,3,4-Methylhippuric acid + phenylglyoxylic acid	Highly stable (identical members)
Pesticide/solvent	2,4-Dichlorophenoxyacetic acid + 3-phenoxybenzoic acid + *N*-acetyl phenylcysteine	2,4-Dichlorophenoxyacetic acid + 3-phenoxybenzoic acid + *N*-acetyl phenylcysteine	Highly stable (members unchanged; component number shifted from PC2 to PC3)
Mitochondrial/fuel	Tiglylglycine + 2-hydroxyisobutyric acid	Tiglylglycine + 2-hydroxyisobutyric acid	Highly stable (members unchanged; component number shifted from PC3 to PC2)
Independent	Monoethylphthalate, diethylphosphate	Monoethylphthalate, diethylphosphate	Stable (unchanged)

## Data Availability

The raw data supporting the conclusions of this article will be made available by the authors on request.
